# Intercostal nerve cryoanalgesia in the treatment of pain in patients operated on by the modified Nuss method with the BackOnFeet application—a new strategy to improve outcomes

**DOI:** 10.3389/fped.2022.1069805

**Published:** 2023-01-12

**Authors:** Sławomir Zacha, Agata Andrzejewska, Barbara Jastrzębska-Ligocka, Aleksander Szwed, Elżbieta Modrzejewska, Wojciech Zacha, Karolina Skonieczna-Żydecka, Jakub Miegoń, Konrad Jarosz, Jowita Biernawska

**Affiliations:** ^1^ Department of Pediatric Orthopedics and Oncology of the Musculoskeletal System, Pomeranian Medical University, Szczecin, Poland; ^2^ Department of Anesthesiology and Intensive Care, Pomeranian Medical University, Szczecin, Poland; ^3^ Department of Orthopedics, Traumatology and Oncology of the Musculoskeletal System, Pomeranian Medical University, Szczecin, Poland; ^4^ Department of Biochemical Science, Pomeranian Medical University, Szczecin, Poland; ^5^ Department of Clinical Nursing, Pomeranian Medical University, Szczecin, Poland

**Keywords:** cryolysis, cryoanalgesia, funnel chest, acute post operative pain, nuss method, cryoanesthesia, ERAS (enhanced recovery after surgery), prehabilitaion

## Abstract

**Introduction:**

The surgical Nuss correction of the funnel chest deformity is a painful procedure without an established consensus of pain relief methods. High doses and long duration of opioids requirements impedes the ERAS protocol introduction. The aim of this study was to evaluate the effectiveness of intraoperative intercostal nerve cryolysis in terms of pain management in relation to the routinely used multimodal analgesia in Poland. We also assessed the impact of using the proprietary “BackOnFeet” application on the quality of life of patients after surgery in relation to the ERAS protocol.

**Methods:**

The prospective, single-centre, non-randomised, before—after pilot study was conducted. Inclusion criteria were: funnel-shaped chest deformity, age range 11–18 years, first chest wall operation, agreement for the cryolysis and regional analgesia, no history of chronic painkillers use. The results of the “control group” (multimodal analgesia with regional analgesia commonly performed in Poland) were assessed. The interdisciplinary perioperative protocol with the “BackOnFeet” application and intraoperative intercostal nerve cryoanalgesia were introduced to the “intervention group”.

**Results:**

Eighteen children were treated with standard protocol typical for Polish management and matched to eighteen patients who received cryoanalgesia and the “BackOnFeet” application access “intervention group”. We noticed lower NRS points in first 24 h (*p* = 0.0048), shortening of time of opioid use (*p* = 0.0002), hospitalisation time (*p* = 0,01), improved quality of postoperative rehabilitation (*p* < 0.0001) and quality of life (*p* < 0.0001) among the “intervention group”.

**Conclusions:**

Intraoperative intercostal nerves cryolysis performed during the minimally invasive Nuss correction of funnel deformation in combination with bilateral is more effective in terms of acute pain management in relation to the routinely used multimodal analgesia in Poland, allowing for the shortening of time of opioid use, hospitalisation time, improved quality of postoperative rehabilitation and enabled ERAS protocol introduction. The use of the proprietary “BackOnFeet” application has a positive effect on the quality of life of patients after surgery.

## GRAPHICAL ABSTRACT

**Figure:**
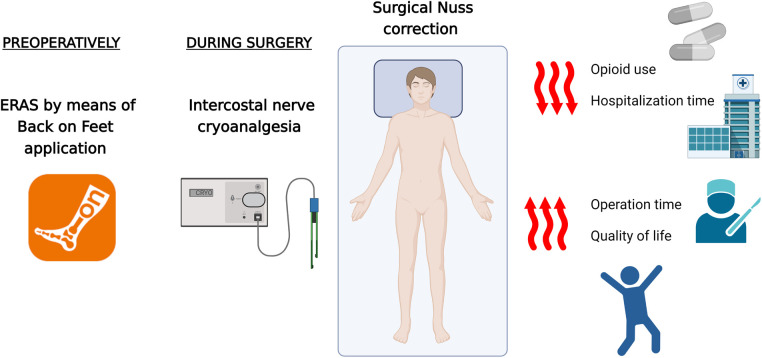
Standarized care with ERAS protocol for NUSS surgery and its benefits.

## Introduction

Deformation of the anterior chest wall (shoemaker, funnel-shaped chest) is the most common congenital deformity of the sternum and the adjacent ribs. It mainly affects boys. Reported prevalence has been 1 in 300 to 1 in 1,000 live births (1). The reason for the occurrence of this defect is disturbances in the endochondral ossification of the sternal parts of the ribs. It is usually diagnosed in the period of the fastest growth spurt and is a cosmetic defect which often causes psychological problems. In the case of high severity of the defect, the proposal of surgical treatment is presented as the only effective method of deformation correction. The currently valid and very effective method is a modified, minimally invasive Nuss method with the use of thoracoscopy (2, 3). In Poland, on average, 500 such procedures are carried out annually. They are mainly performed by pediatric and thoracic surgeons. The chest surgery causes pain of significant intensity. However, there is no established consensus on pain relief methods. On average, severe pain lasts up to several weeks after the surgery (4). Apart from the use of opioids and sedatives, the current standard of care has been regional analgesia (usually placement of an epidural catheter in the thoracic section for 3–5 days, intrapleural analgesia with intercostal nerves block or paravertebral block) (5–10). It is connected with the necessity of prolonged hospitalisation in order to control severe pain, so early rehabilitation is difficult. There is also a risk of side effects due to pharmacotherapy and the use and maintenance of an epidural catheter (hematoma, neurological disorders in the lower extremities, toxicity of local anesthetics and urinary retention) (6). After removal of the catheter and the end of drug delivery to the epidural space, severe pain recurs. Despite the use of multimodal analgesia, the intensity of pain in the postoperative period is significant, the duration of pain is long, and the risk of chronic pain is high (4). Therefore, additional methods of pain relief are still being sought, which, in combination with the standards in force so far, could improve the patient's comfort and safety.

### Cryoanalgesia

The treatment of acute pain by means of blockage of nerve conduction due to the use of low temperatures has been known since the time of Hippocrates. The modern version of “freezing nerves” with the use of dedicated probes uses the Joules-Thomson effect, i.e., a sudden change in gas temperature (nitrogen oxide or carbon dioxide) during its expansion as it passes through a porous partition from a place of higher pressure to an area of lower pressure. The tip of the probe suddenly cools down to a temperature of −50 to −80 °C. Adjacent tissues, including nerves, are exposed to a temperature of around −30 °C, which causes the degeneration of axons and myelin sheaths (Wallerian degeneration), temporarily blocking the nerve conduction and, preferably, the pain sensation (11, 12). The endoneurium, perineurium and epineurium remains intact, which facilitates regeneration, which takes about 4–6 weeks or even several months (11). The nerve is rebuilt at the rate of 1 mm/week, and the return of sensory functions takes place between 3 and 6 months. There is no risk of neuroblastoma formation (13). It can therefore be assumed that the time of experiencing severe pain after Nuss's surgery coincides with the blockage of conduction in intercostal nerves “frozen” by cryolysis. The treatment may be repeated. The expected benefits of using cryoanalgesia in the correction of chest deformity in relation to standard treatment include the reduction of the severity of postoperative pain, the reduction of the risk of persistent pain, and the reduction in the need for analgesics used in the postoperative period. This translates into the possibility of shorter hospitalisation and reduction of the total cost of treatment. In the available literature, the results of clinical trials confirm these assumptions, but there are still reports in a small number of cases (4–10) The AtriCure surgical system (AtriCure, Inc., West Chester, Ohio) was used in the most of these cases.

The device of the Polish company Cryo-S Painless and the A-30/300/PEA/RF probe (Metrum Cryoflex, Poland) are dedicated to performing intraoperative cryoanalgesia during thoracoscopy. In Poland, up till now, the cryoanalgesia method has been used to relieve pain only in adults. In May 2022, the procedure of intraoperative cryolysis of the intercostal nerves during minimally invasive modified Nuss correction was performed in children for the first time in Poland at the Department of Pediatric Orthopedics and Oncology of the Musculoskeletal System of the SPSK No. 1 of the Pomeranian Medical University in Szczecin.

### Prehabilitation

Prehabilitation, understood to be the application of appropriate physical exercises and nutritional support before the surgery, is an effective strategy combining actions taken by the patient, family and therapeutic team in order to achieve the best possible effect of the surgery. The initiation of prehabilitation while waiting for the planned procedure along with the use of a comprehensive perioperative care protocol to improve treatment outcomes (ang. enhanced recovery after surgery, ERAS) enable faster recovery, greater patient and family satisfaction, and shorter hospitalisation time by reducing the risk of complications (14). The key aspect is proper patient and family education. There is usually not enough time for this during preoperative visits to the clinic. That is why our interdisciplinary team created a free educational and training application “BackOnFeet” for patients and families to optimise their activities while waiting for the surgery.

The aim of this study was to evaluate the effectiveness of intraoperative intercostal nerve cryolysis performed during the correction of funnel-shaped deformation of the anterior chest wall with the minimally invasive Nuss method in terms of acute pain management in relation to the routinely used multimodal analgesia in Poland. The duration of opioid use, length of hospitalisation and the quality of rehabilitation were also assessed. An additional goal was to assess the impact of using the proprietary “BackOnFeet” application on the quality of life of patients after surgery.

## Methodology

A prospective, single-centre, non-randomised, before—after pilot study was conducted. The study included 36 teenagers undergoing minimally invasive modified Nuss elective surgery due to funnel-shaped deformation of the anterior chest wall. Exclusion criteria were: chest deformity other than funnel-shaped, advanced chronic respiratory-circulatory failure, emergency surgery, reoperation, previous thoracotomy or thoracic surgery, mental impairment preventing communication with the patient, refusal to consent to regional anaesthesia, refusal to perform cryolysis, history of chronic painkillers use. All study participants over 16 years of age and their legal guardians gave informed consent to participate in the study. The study protocol was registered under the NCT 05570097 at clinicaltrials.gov. The approval number of the bioethics committee KB-006/43/2022.

The study was designed to compare the standard therapy in the “control group” (multimodal analgesia with regional analgesia commonly performed in Poland) to the results of the “intervention group”, in which the intraoperative cryoanalgesia procedure was added to the current multimodal and regional analgesia. Additionally, this group had an interdisciplinary perioperative protocol in the form of access to the “BackOnFeet” application. The “control group” consisted of 18 patients (mean age 15 years), 17 boy with standard protocol. Its results were analysed, and based on them, the protocol of the “intervention group” was established. The “intervention group” consisted of 18 patients (mean age 14), 17 boys.

### Preparation for surgery

After the surgery was scheduled, the patients were referred for an anesthetic consultation and then qualified for general and regional anesthesia. Before the surgery, a questionnaire was conducted with the patient and the parent according to the modified Nuss questionnaire (15). It is a two-step questionnaire for a paediatric patients (including questionnaires for both patients and their parents) which assesses the effect of the surgical procedure on the psychosocial and physical functioning of the patients. The questionnaires included 12 items for patients and 13 items for parents (about the patients) with values between 1 and 4.

On the day of the procedure, as an preemptive analgesia, the patient received metamizole (15 mg/kg for children under 50 kg or 1 g orally if the body weight exceeds 50 kg), along with a carbohydrate-rich fluid (Preop, Nutricia Poland) administered orally two hours before the surgery.

### Anesthesia

All patients were subjected to general anesthesia with intubation with a double-lumen tube and mechanical ventilation with the Primus apparatus (Draeger, Germany). After induction for general anesthesia (fentanyl 2–4 mcg/kg, propofol 3–5 mg/kg, ketamine 0.5 mg/kg, sevoflurane 1–1.3 MAC) the patients were administered drugs according to the principles of multimodal analgesia, including regional analgesia—bilateral one time intrapleural blockade or erector spinae plane block—ESP block (according to NYSORA's Nerve Blocks, USA) with the use of 0.25% bupivacaine (total dose less per 0.5 mg/kg). Drugs administered intravenously during the procedure: fentanyl (2–4 mcg/kg), acetaminophen (15 mg/kg for children under 50 kg or 1 g intravenously if the body weight exceeds 50 kg), ibuprofen (10 mg/kg for children under 40 kg or 400 mg intravenously if body weight over 40 kg), magnesium sulfate (50 mg/kg as an intravenous infusion), dexamethazone (0.15–0.5 mg/kg), ketamine (0.1–0.2 mg/kg boluses every hour), ondansetron (0.15 mg/kg).

Maintenance of general anesthesia with sevoflurane and fentanyl, ventilation of one lung, expansion, selective ventilation of the other lung, expansion. Reversal of neuromuscular block with Sugammadex 2 mg/kg and recovery was performed in the standard manner. Approx. thirty minutes before the end of the procedure, an opioid bolus was administered: morphine <12 years old or oxycodone >12 years old, at a dose of min. 100 mcg/kg followed by a continuation of the infusion of 10–40 mcg/kg/h in an infusion pump.

### Operation

After qualifying for surgery based on a physical examination and computed tomography, all patients underwent minimally invasive correction surgery using the modified Nuss technique with the use of thoracoscopy (1, 2). All surgeries and cryoanalgesia were performed by a single pediatric orthopedic specialist with extensive prior experience in treatment of thoracic deformities. In the supine position both upper limbs were placed in abduction around 70 degrees to ensure full operational access to the chest, while preventing damage to the brachial plexus. The appropriate length of the plate was selected and bent according to the size and shape of the chest. The place of insertion of the plate into the pleural cavity was marked with a marker in the most prominent intercostal spaces on both sides of the chest cavity. Bilateral skin incisions were made in the anterior axillary lines transversely to the long axis of the body. Two thoracoscopy ports were then made in the nipple lines approximately 4 cm below the skin incisions and a plate was placed under the sternum. The last stage of the procedure was the rotation of the previously introduced plate by 180 degrees. During this manoeuvre, the rotating plate lifted the sternum with the adjacent deformed parts of the ribs. Both ends of the plate rested on ribs in the front armpit lines. To prevent secondary displacement of the implant, stabilising plates were placed on one side. Additionally, both ends of the plate were attached to the ribs with Ethibond Exel (Ethicon, Johnson & Johnson, USA) insoluble thread. Before the layered suturing of the operating accesses, a thoracoscopic inspection of the plate passage under the sternum was performed in order to exclude bleeding.

### Postoperative course

After surgery, all patients were followed in the post-anesthesia care unit (PACU) for 24 h. In addition to the assessment of vital signs, a pain intensity assessment protocol according to the numerical scale (NRS) was performed every hour for 24 h, and then every 8 h until discharge from the hospital with the type, dose and route of administered drugs, the occurrence of side effects and complications. In the postoperative period, drugs were administered at regular intervals: acetaminophen (15 mg/kg below 50 kg or 1 g if the body weight exceeds 50 kg), orally or intravenously every 6 h, metamizole (15 mg/kg below 50 kg or 1 g if the body weight exceeds 50 kg), orally or intravenously every 6 h, ibuprofen (10 mg/kg less than 40 kg or 400 mg if the body weight exceeds 40 kg), orally or intravenously every 8 h, magnesium sulphate 35–50 mg/kg intravenous infusion every 12 h for 48 h, morphine infused 10–40 mcg/kg/h in an infusion pump and boluses max 0.1 mg/kg every 4 h, if resting NRS exceeded 3 points or in exercise > 6 points. Intravenous opioid infusion and conversion to oral treatment were determined according to the daily requirement.

One month after the procedure, the patient and parent questionnaire was repeated according to the modified Nuss questionnaire, and the occurrence, intensity of pain and disturbances in chest sensation were assessed.

The described scope of preoperative preparation, the technique of Nuss surgery, the type of analgesia used in the perioperative period, and the parameters assessed after the procedure were the same in the “control group” and “intervention groups”. In both groups, the hospitalisation plan was based on the principles of the enhanced recovery after surgery (ERAS) protocol. The current definition of ERAS program includes a multidisciplinary approach to improve surgical outcomes by using procedure-specific evidence-based protocols in the care of surgical patients.

### Intervention

The “intervention group”, after setting the date of the procedure, had access to the proprietary educational and training application “BackOnFeet”. Thanks to the app, patients could start prehabilitation at home as preparation for surgery (free access in Google Play, Apple Store, information at: www.backonfeet.pl).

Intercostal nerve cryolysis was performed intraoperatively with the use of the Metrum Cryoflex Cryo-S Painless device. A specially shaped probe dedicated to intraoperative cryolysis was inserted into the pleural cavity in the same intercostal space as the previously inserted plate using a cut skin incision. Appropriate visualisation conditions were ensured by ventilation of one lung on the opposite side at that time. Routine cryolysis was performed on 5 levels (2 above, 2 below and at the level of plate insertion, usually Th3-Th8). In the case of extensive deformities, the number of intercostal nerves subject to freezing was greater (max 7). A properly bent probe tip was applied below the lower edge of the rib laterally from the transverse processes prior to the departure of the connecting branches from the intercostal nerves. The time of a single application of cryolysis was two minutes each time per intercostal nerve. A similar procedure was performed symmetrically on the other side. In total, the cryoanalgesia procedure lasted about 25–30 min.

### Definitions of complications and adverse drug reactions

Adverse drug reactions were defined as: nausea, vomiting, pruritus, breathing difficulties, constipation, urinary retention, dizziness, somnolence preventing rehabilitation, apnoea, hypotension depending on physiological values typical for age, bradycardia and a decrease in SpO2 < 90%.

Complications of the cryolysis procedure were defined as: neuropathic-specific pain described as “burning” or “tingling” sensations in the operated area.

Complications of Nuss correction were defined as: respiratory-circulatory failure, pneumothorax requiring drainage, haematoma, surgical site infection, pleural empyema, pericarditis, bar dislocation requiring reoperation, pain impossible to relieve in a standard way, pneumonia, cardiac perforation, death.

The evaluation of the obtained results was a comparison in terms of:

– demographic data,
– evaluation of acute pain intensity (maximum) in the first day after surgery—measurement every hour for 24 h using the numerical scale of the NRS (0–10 points),
– the duration of intravenous opioid use (up to which day after surgery),
– the quality of postoperative rehabilitation in terms of the correctness of the exercises performed and achieving motor independence in everyday activities (from what day after the surgery),
– duration of the surgical procedure “skin to skin” (minutes), duration of hospitalisation (days), analysis of hospitalisation costs,
– quality of life before and after the procedure assessed according to the modified Nuss questionnaire (range of changes in the score obtained in the survey),
– side effects after opioids,
– the occurrence of complications after cryolysis and Nuss surgery,
– usefulness of the “BackOnFeet” application was assessed in the questionnaire: Was there information about the procedure? Did the child follow the dietary recommendations and perform the recommended physical exercises regularly? What was the general assessment of the usefulness of the application (0–5 scale)?

### Statistical analyses

The following methods were used to analyse the results. Shapiro-Wilk normality test was done to assess the distribution of continuous variables. Nonparametric Mann-Whitney test was used to compare variables in study group and control subjects. Paired Wicoxon test was used to see the difference in patients' and parents' quality of life after the surgery. To analyse the link between qualitative variables, chi2 test was used. The acceptable probability of error for the first type was assumed to be equal to 0.05. MedCalc statistical software version 20.110 (Ostend, Belgium).

## Results

A total of 36 patients underwent a minimally invasive modified Nuss procedure for a pectus deformity between January and September, 2022. Eighteen patients underwent multimodal analgesia with regional analgesia (intrapleural analgesia of intercostal nerves) after the Nuss procedure. The results were analysed and interdisciplinary protocol was prepared. Before operation eighteen patients who met the inclusion criteria to the study, called “intervention group”, did a protocol (prehabilitation with the application “BackOnFeet”, ERAS protocol perioperatively). During the operation this group underwent multimodal analgesia with regional analgesia (ESP block) and cryoanalgesia of the intercostal nerves during thoracoscopy. A comparative analysis of the preoperative data obtained from patients in the “intervention group” and the “control group” is presented in Table 1.

**Table 1:** Demographic data from patients in both groups.

Parameters	Control *n* = 18	Intervention *n* = 18	*p*
Age	14 (13–15)	15 (11–16)	ns
Gender (boys/girls)	17/1	17/1	ns
BMI^a^	19,5 (16–19)	18 (17–20)	ns
ASA^b^ 1	17	17	ne
ASA^b^ 2	1	1	ne
ASA^b^ > 2	0	0	ne

^a^ BMI, body mass index.

^b^ ASA, American society of anaesthesiology, ns, non significant; ne, non estimable; quantitative data: median and IQR.

The surgery data including hospitalization time and rehabilitation are presented in Table 2.

**Table 2:** Surgical data from patients in both groups.

Parameters	Control *n* = 18	Intervention *n* = 18	*p*
Operation time (minutes)	60 (60–70)	90 (70–90)	0,0013
MAX NRS 1^a^	5 (2–5)	3 (4–6)	0,0048
OPIOIDS^b^	2 (2–3)	1 (1–2)	0,0002
Proper quality of rehabilitation (day)	3 (2–4)	1 (1–2)	<0,0001
Independence^c^ (day)	6 (5–6)	2 (2–3)	<0,0001
Hospitalisation time (days)	6 (5–6)	4 (4–5)	0,0119

^a^ MAX NRS 1- value of acute pain intensity (maximum) on the first day after surgery according to the NRS scale.

^b^ OPIOIDS—the day of termination of intravenous opioid use, Independence.

^c^ A day of full independence during daily activities, quantitative data: median and IQR.

We noticed the reduction of total hospitalisation costs in the “intervention group” due to a shorter hospitalisation time (*p* = 0.407). However, the costs of used material in this group were significantly higher (*p* = 0.0015).

All of the patients were assessed at the discharge and one month after discharge in the outpatient clinic. The postoperative data including complications and survey are presented in the Table 3.

**Table 3:** Postoperative data from patients in both groups.

Parameters	Control *n* = 18	Intervention *n* = 18	*p*
Child survey (after-before change)^a^	20	39	<0,0001
Parents survey (after-before change)^a^	27	50	<0,0001
Side effects of opioids^b^	6	4	ne
Complications after Nuss surgery^b^	1	2	ne

^a^ Points.

^b^ Number of patients. ns, non significant; ne, non estimable.

**Figure 1:**
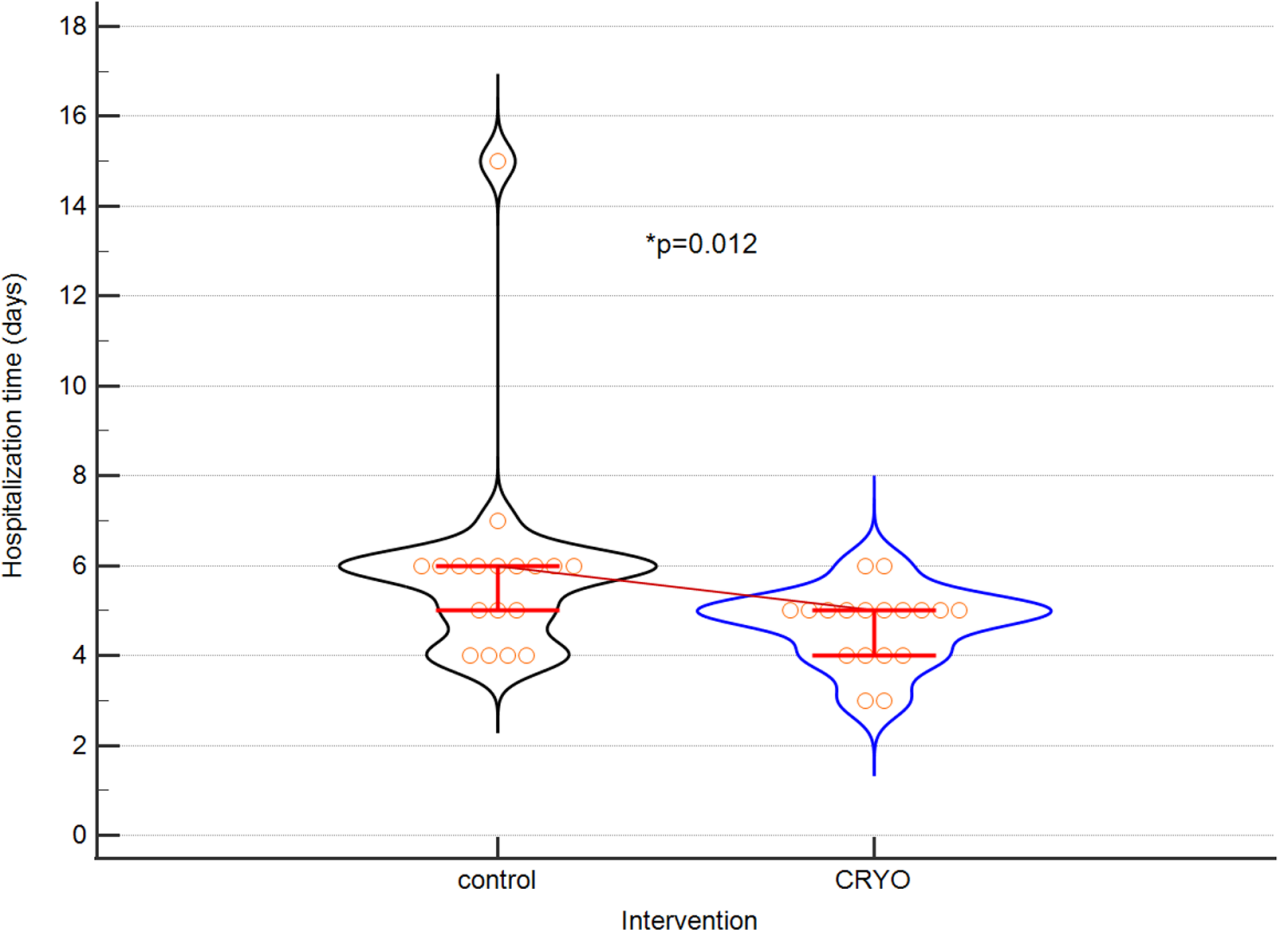
Course of the study.

**Figure 2:**
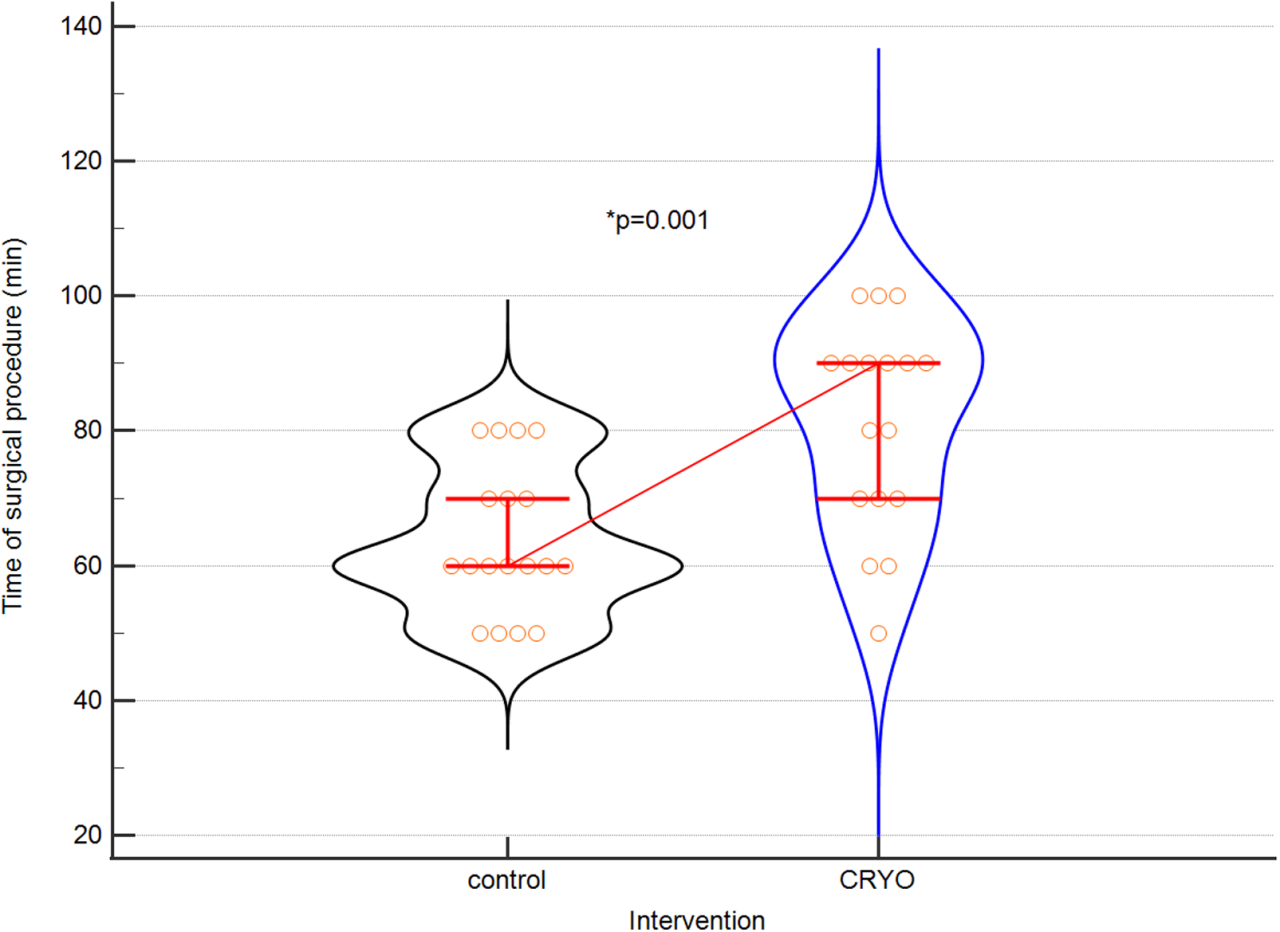
The difference between hospitalization time of both groups. Violin plot for hospitalization time. Medians and IQRs are shown. Circles depict individual cases. CRYO means interventional group.

The results of the difference of hospitalization time are presented on Figure 1. Figure 2 depicted the difference of surgical procedure time.

In one patient, hypoaesthesia of the part of the skin in the area of the sternum was a possible complication after the cryolysis procedure for several days. The disorder resolved spontaneously.

## Discussion

The presented study is the first report which evaluated the effectiveness of the intraoperative cryoanalgesia of the intercostal nerves by a dedicated curved, golden tip Cryo-S Painless probe (Metrum Cryoflex, Poland) during the correction of funnel-shaped deformation of the anterior chest wall using the minimally invasive Nuss method in the pediatric population. This type of probe was first time used for children in Poland. Previous type of straight probe was used in Italy, Spain, Argentina (16). The technical modification of the probe facilitates positioning. Our study confirmed the effectiveness of cryoanalgesia in terms of better pain control after surgery, the possibility of shortening the hospitalisation time, shorter duration of intravenous opioid use, improving the quality of rehabilitation and quality of life of patients in relation to the routinely used multimodal analgesia in Poland. A recent series of studies in pectus excavatum patients demonstrated lower pain scores, opioid use, and hospitalization among patients receiving cryolysis (17–25). In addition, we confirmed the beneficial effect of the use of the proprietary “BackOnFeet” application on the comfort of patients after surgery. This study presents the results of the first Polish application of intraoperative cryolysis of intercostal nerves.

The challenge during thoracic surgery is to control severe pain. Initial reports comparing the effectiveness of continuous intravenous opioid administration with the use of an epidural catheter compared to the effectiveness of cryoanalgesia during thoracic surgery procedures emphasised the advantage of opioids and a high risk of cryolysis complications up to 30% of long-term neuralgia (26). Descriptions of the first cases of using cryolysis in thoracic surgery before 2015, and then the results presented by the research group Kim et al., in which a modified technique of probe access to the opposite intercostal nerves through the anterior mediastinum was used, were controversial, so this method was reluctantly used (17). Subsequent studies indicated the possibility of more effective pain control and shorter hospitalisation (4, 18–25). They were case series descriptions used the AtriCure surgical system. The principle of cryolysis has become an attractive alternative in the possibilities of analgesic treatment. Therefore, the equipment and technique of the procedure were improved. The research group of Graves et al. has shown that the use of selective lung ventilation during cryolysis improves visualisation during thoracoscopy and allows the probe to reach the site before the collaterals depart from the intercostal nerves near the transverse processes of the vertebrae, which is considered more effective (4, 18–25, 27). It was also used in our study.

Despite the fact that the studied groups of patients were few and the type of regional anesthesia protocol used during multimodal analgesia differed (continuous block through epidural anaesthesia, bilateral single-shot ESP block, intrapleural blockade of intercostal nerves), each time the authors emphasised that significantly better results were obtained when the cryolysis procedure was added to the standard procedure (4, 18–25, 27). In terms of the impact on the effectiveness of acute pain relief and the resulting reduction of the total dose and the duration of intravenous opioid use, the results of our study are consistent with those described so far in the literature.

The beneficial effect of the use of cryoanalgesia by reducing the amount of opioids used also translates into a reduction in the occurrence of side effects after pharmacotherapy and regional analgesia using an epidural catheter (4, 18–22). The use of cryolysis is therefore a safer alternative in relation to the need to maintain the catheter in the epidural space for several days.

In view of the reports in the literature about the possibility of using cryolysis both during surgery and percutaneously—before the procedure, the question arises which of the methods brings more benefits (21–25, 27–29). The intraoperative technique performed by the operator during thoracoscopy has undoubted advantages of direct contact and visual inspection. The disadvantage is the extension of the time of the procedure and the patient's stay in the operating theatre. The percutaneous technique of the procedure enables it to be carried out before the operation at any time in advance. However, it is associated with the need to perform additional general anaesthesia in the patient and non-optimal control of the probe position by means of ultrasound (indirect visualisation) (29). The time to reach full effectiveness of cryoanalgesia is currently unresolved. Hence the concepts of percutaneous nerve freezing under ultrasound control 12–48 h in advance before thoracic surgery (28, 29). Our experience shows that the solution may be the use of preoperative regional analgesia (bilateral ESP block) “overlap” with intraoperative cryolysis. However, more studies on a larger group is needed.

Other debatable issues include the timing of full nerve regeneration, the incidence of neuropathic pain, and the role of gabapentinoids in securing proper nerve block and preventing neuropathic pain. The intraoperative intercostal nerve cryolysis procedure itself is a safe procedure. The following complications are described in the literature mainly in adults: persistent neuropathic pain, hyperaesthesia, temporary pain in the operated area, bleeding from a wound or deep structures, lung damage, clinically significant pneumothorax, haematoma (20). Graves et al. reported persistent numbness up to several months in two children undergoing Nuss procedure (4). Burdant et al. described infectious complications of intercostal nerve cryoablation mediated by perioperative hypothermia during pediatric Nuss procedure (30). The exact rate of complications in the pediatric population is unknown because of a small rate of reported patients with the cryolesia procedure. In our study population, apart from the occurrence of a slight transient hypoaesthesia in the chest, no complications were observed.

The fact of using cryoanalgesia during surgery is associated with the need to extend the time of the surgical procedure. Even the shortest version of this procedure, i.e., bilateral cryolysis, five intercostal nerves for two minutes for each application, will extend the procedure by at least twenty minutes. Including the preparation of the equipment, the technical optimisation of the probe application and the expansion of the lung during the change of the operated side requires an additional 10–20 min. The average prolongation of the door-to-door time in the literature is approx. 30–45 min and our results do not differ significantly from those obtained by other researchers (18–25, 27, 28).

The duration of hospitalisation described in the literature is significantly shorter in patients undergoing cryolysis compared to the “control group” (4,18–25, 27–29). This fact was confirmed in our study. This translates directly into a significant reduction in the total cost of hospitalisation.

The quality of postoperative rehabilitation in terms of the correctness of the exercises performed and the time to achieve motor independence in everyday activities is better in the group subjected to intervention (cryolysis procedure). Effective pain control and the lack of a sedative effect of opioids enable the patient to both effectively rehabilitate and quickly regain independence. Additionally, the knowledge of breathing exercises and the type of postoperative rehabilitation trained while waiting for the procedure enhances this effect. Therefore, the “BackOnFeet” application prepared by our interdisciplinary educational and training team improves and accelerates recovery, gives the patient a sense of influence on their disease and the recovery process, which translates into an enthusiastic reception. The “BackOnFeet” application facilitates educational process which is key aspect of ERAS protocol (14, 20). We suggest to use our “BackOnFeet” application in the future studies.

Chest deformities are mainly a psychological problem, less often a functional one. The appearance is the main reason for the decision to undergo surgery, as the quality of life before surgery is assessed by patients as low. The modified Nuss questionnaire dedicated to patients with chest deformities is a useful and commonly used tool to assess the quality of life of patients and their families (15, 31, 32). In our study, we assessed patients' quality of life before and after surgery using this questionnaire. We showed a significantly greater range of changes in the scores obtained in the survey.

## Conclusions

Intraoperative cryolysis of the intercostal nerves performed during the correction of funnel deformation of the anterior chest wall using the minimally invasive Nuss method in combination with bilateral ESP blockade is more effective in terms of acute pain management in relation to the routinely used multimodal analgesia in Poland, allows for the shortening of the time of opioid use, hospitalisation time, and improved quality of postoperative rehabilitation. In addition, the use of the proprietary “BackOnFeet” application has a positive effect on the quality of life of patients after surgery.

## Data Availability

The original contributions presented in the study are included in the article/Supplementary Material, further inquiries can be directed to the corresponding author/s.
